# Hydrogen Sulfide: A Worthwhile Tool in the Design of New Multitarget Drugs

**DOI:** 10.3389/fchem.2017.00072

**Published:** 2017-09-27

**Authors:** Simona Sestito, Giulia Nesi, Rongbiao Pi, Marco Macchia, Simona Rapposelli

**Affiliations:** ^1^Department of Pharmacy, University of Pisa, Pisa, Italy; ^2^School of Pharmaceutical Sciences, Sun Yat-Sen University, Guangzhou, China; ^3^International Joint Laboratory (SYSU-PolyU HK) of Novel Anti-Dementia Drugs of Guangdong, Guangzhou, China; ^4^Guangdong Province Key Laboratory of Brain Function and Disease, Zhongshan School of Medicine, Sun Yat-sen University, Guangzhou, China

**Keywords:** hydrogen sulfide, multitarget-directed ligands, bone disease, hybrid molecules, H_2_S-donors, multifunctional compounds, antioxidants, neurodegenerative disorders

## Abstract

H_2_S is a gaseous molecule able to trigger a plethora of central physiological and pharmacological effects as antioxidant, pro- and anti-inflammatory, pro- and anti-nociceptive, neuromodulator, and cytoprotective. The polypharmacology of H_2_S depends on the wide variety of targets implicated, but, despite the efforts, the mechanisms of action that should clarify its activity are still not completely unrevealed. Nevertheless, many attempts to exploit the multifaceted profile of this molecule have already been accomplished and many chemical entities containing an H_2_S-releasing pharmacophore have been synthetized. Here we discuss recent investigations on multitarget molecules able to release H_2_S, with a particular focus on the combinations of “native drug” with moieties structurally able to release H_2_S and their applications as therapeutic tools in bone disease, gastrointestinal system and neurodegenerative disorders.

## Introduction

Nitric oxide (NO), carbon monoxide (CO) and more recently hydrogen sulfide (H_2_S) have emerged as “gasotransmitters” with central physiological and pharmacological effects. In the past three decades, NO has been widely investigated for its role in controlling blood circulation and regulating activities of the brain, lungs, liver, kidneys, stomach and other organs (Marsh and Marsh, 2000). More recently, H_2_S, another “toxic gas,” also appeared as important regulatory mediator (Moore et al., 2003).

H_2_S and NO exhibit many common traits like the ability to cross the biological membranes and to penetrate cells without the need of specific membrane receptors. Moreover, these molecules have been involved in the induction of hippocampal long-term potentiation, thus suggesting a key role as neuromodulators in the brain (Qu et al., 2008). Additionally, both mediators are well-known for their capability to regulate the blood pressure both *in vitro* and *in vivo* (Ali et al., 2006).

H_2_S plays the same multiple protective roles in the vascular system exhibited by NO but, in contrast to this latter, H_2_S is not associated with the harmful production of reactive oxygen species (ROS) (Whiteman et al., 2004). Probably H_2_S protects against cellular damage through an up-regulation of the nuclear-factor-E2-related factor-2 (Nrf2)-dependent signaling pathway (Calvert et al., 2009, Figure 1).

**Figure 1:**
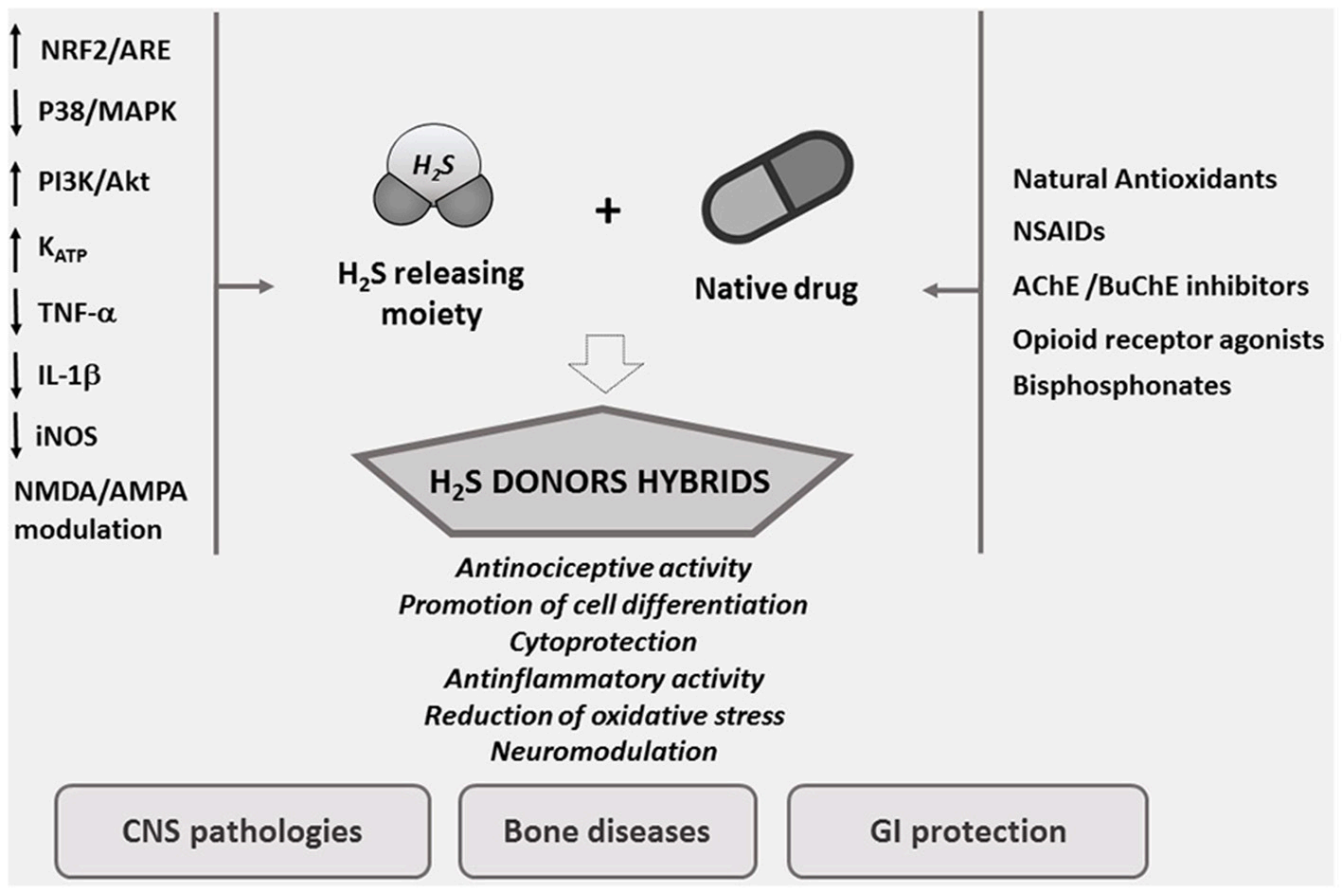
Designing smart agents through the combination of H_2_S pharmacological properties with old drugs.

In mammals, enzymatic H_2_S biosynthesis originates primarily from cysteine and homocysteine metabolism due to cystathionine-γ-lyase (CSE), cystathionine-β-synthase (CBS), and 3-mercaptopyruvate sulfurtransferase (3-MST)/cysteine aminotransferase (CAT) (Kimura, 2010). The expression of these enzymes is tissue-specific, with CBS being found predominantly in the brain and nervous system, while CSE is mainly expressed in the liver and in vascular/non-vascular smooth muscle. Conversely, 3MST is ubiquitously localized in many systems such as liver, kidney, heart, lung, thymus, testis, thoracic aorta and brain (Shibuya et al., 2009a,b).

The rapidly expanding literature related to H_2_S shows that this gasotransmitter triggers cardiovascular protection (Sun et al., 2017b; Wallace et al., 2017), possesses antitumor activity (Ianaro et al., 2016), regulates ion channels function (Naik et al., 2016; Lu et al., 2017), and displays antioxidants effects (Egea et al., 2015). Given these physiological roles, H_2_S appears to act as a mediator in several functions, thus representing a potential therapeutic agent as well as NO and CO.

## Hydrogen sulfide and effects on bone

Recent studies on physiological and pathophysiological roles of H_2_S, revealed its therapeutic potential in bone diseases (Liu et al., 2014; Zhai et al., 2017).

Osteoporosis is an endemic bone disease of the Western society characterized by an imbalance between bone resorption and bone formation. Physiologically, osteoblasts are responsible for bone formation and osteoclasts are associated with bone degradation. Although the exact mechanism linking H_2_S to bone formation is still not clarified, it turned out that this gasotransmitter inhibits the differentiation of osteoclasts through a mechanism involving antioxidant response without affecting cell viability (Gambari et al., 2014). Indeed, the study of Xu et al. showed that H_2_S protects osteoblastic MC3T3-E1 cells against oxidative stress (OS) via inhibition of mitogen-activated protein kinase (MAPK) signaling (Xu et al., 2011). Interestingly, H_2_S-donors such as GYY4137, stimulates osteogenic differentiation of human mesenchymal stromal cells (h-MSCs) both *in vitro* (Grassi et al., 2016) and *in vivo* (Liu et al., 2014). Consequently, the dual activity profile of H_2_S inspired the development of new molecules based on the multitarget-directed ligands (MTDLs) approach as alternative strategy to design new potential treatments for bone diseases.

The traditional Chinese medicine Danshensu, also known as DSS (3-(3,4-dihydroxyphenyl) lactic acid), is a natural phenolic acid isolated from *Salvia miltiorrhiza* root, recognized for its ability to reduce inflammation and suppress ROS formation (Lu et al., 2014; Jiang et al., 2015). In a more recent study, it turned out that DSS protects bone from glucocorticoids-induced bone marrow impairment through the stimulation of osteogenesis; moreover, it depresses adipogenesis in bone marrow stromal cells both *in vivo* and *in vitro* (Liao et al., 2009). These observations prompted Bian to develop a multifunctional molecule starting from acetyl-DSS and 5-(4-hydroxyphenyl)-3H-1,2-dithiole-3-thione (ADT-OH), a well characterized molecule known to release H_2_S (Yan et al., 2017). It has been demonstrated that the newly synthesized hybrid, SDSS (α-3, 4-tris (acetyloxy) benzenepropanoic acid 4-(3-thioxo-3H-1,2-dithiol-5-yl)phenyl ester) (Table 1), after injection into animals, is quickly deacetylated or de-esterified generating ADT-OH, an intermediate capable to induce H_2_S-release both *in vivo* and *in vitro*. The H_2_S release from SDSS has been confirmed through a fluorescent probe in MC3T3-E1 cells. The new compound also prevents the reduction of the alkaline phosphatase activity, the loss of collagen expression, and the inhibition of bone nodule formation in osteoblasts treated with H_2_O_2_. Moreover, SDSS seems to suppress OS and improve mitochondrial function in MC3T3-E1 cells, as well as to inhibit MAPKs and activate the phosphatidylinositol 3-kinase/Akt pathway (Yan et al., 2017). These results suggest that SDSS may protect osteoblasts from OS-induced cell injury and stimulate cell differentiation.

**Table 1:** Structure of MTDL compounds and their *in vitro* and *in vivo* H_2_S-mediated biological effects.

**Structure**	**Chemicals name**	***In vitro* H_2_S-mediated effects**	***In vivo* H_2_S-mediated effects**
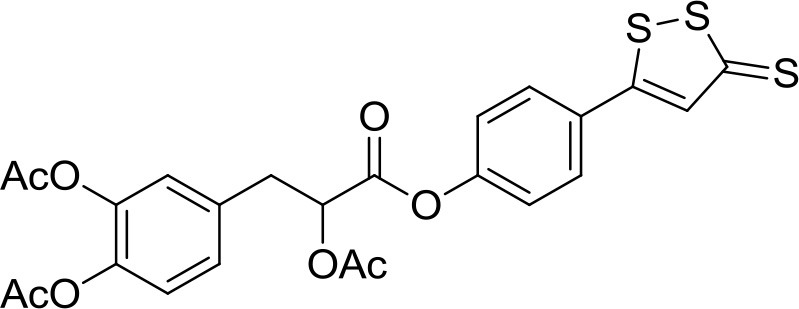	SDSS	Protection of osteoblasts from OS	n.a.
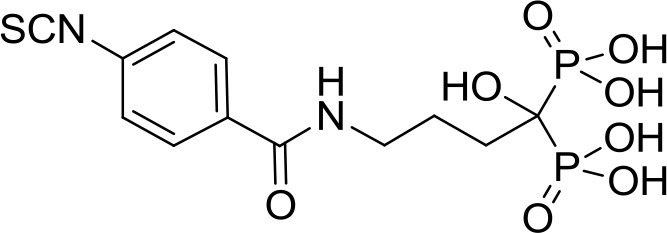	DM22	Increase of both anabolic and anti-resorptive functions	n.a.
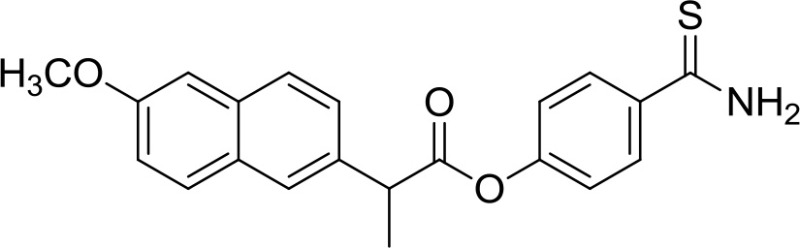	ATB-346	n.a.	Gastroprotection
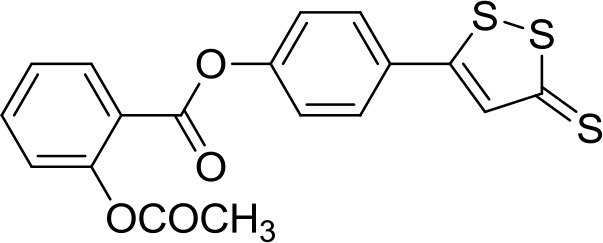	ACS14	n.a.	Preservation of gastric mucosa integrity
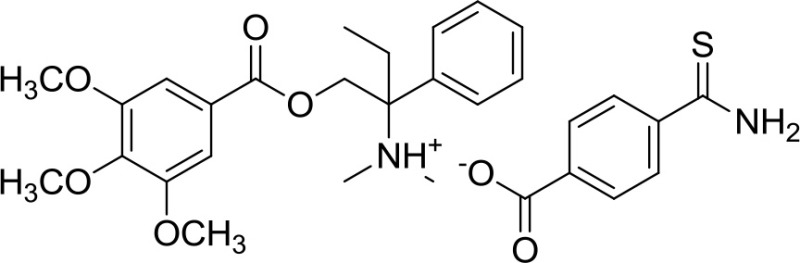	GIC1001	n.a.	Reduction of nociceptive response to all injuries
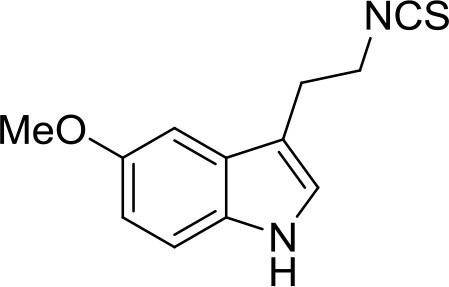	ITH12674	Induction of neuroprotective antioxidant properties and enhancement of the Nrf2–ARE transcriptional response	n.a.
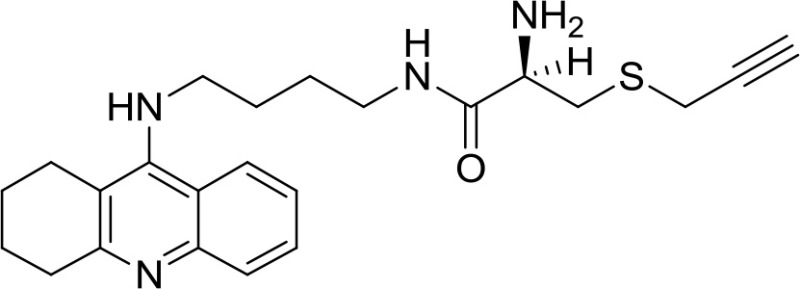	Tacrine-SPRC	Prevention of Aβ-induced toxicity and OS	n.a.

*n.a., not available*.

Nowadays, bisphosphonates (BPs) still represent the first-line and the most prescribed drugs in the treatment of osteoporosis and in the management of metastatic bone disease. BPs have a relatively good safety profile and are tolerated by the majority of patients even though they show several adverse effects among which: gastrointestinal injury, osteonecrosis of the jaws (ONJ) and atypical subtrochanteric femoral fractures (Conte and Guarneri, 2004; Ruggiero, 2011; Kharazmi et al., 2014).

In order to overcome the side effects of BPs, Rapposelli et al. have recently synthesized an innovative molecule called DM-22 (Table 1), obtained by the combination of alendronate (AL) with aryl-isothiocyanate moiety as H_2_S-releasing group (Lisignoli et al., 2016). Amperometric measurements of H_2_S generation from DM-22 showed that the hybrid compound is able to release H_2_S in the presence of organic thiols, such as L-cysteine, with a long-lasting kinetic. Comparing DM-22 with the progenitor molecule (i.e., AL), a substantially improved profile was observed in terms of safety and efficacy on human mesenchymal cell lines (h-MSCs) induced to osteogenic differentiation. Contrary to AL, this new molecule is also able to induce a nearly 3-fold increasing in the mRNA expression of Collagen I and Bone Sialoprotein (BSP) compared to control MSC, thus leading to bone mineralization. This multitarget compound showed, in bone cells *in vitro*, an increase of both anabolic and anti-resorptive functions compared to the parent drug. In the light of these findings, DM-22 could represent a prototype of a novel family of hybrid molecules useful in treating bone loss.

## Hydrogen sulfide and effects on gastrointestinal system

H_2_S is closely linked with the gastrointestinal (GI) system and it is also implicated in its regulation (Bala et al., 2014; Brzozowski et al., 2017; Souza et al., 2017). Beside the host production, H_2_S can be produced exogenously during the digestive processes or by microbes. Its pro- and anti-inflammatory, smooth muscle relaxant, pro-secretory, and pro- and anti-nociceptive actions are well documented in literature, even though its activity is not clarified as a whole (Linden, 2014).

H_2_S is involved in the cytoprotection of gastric mucosa; gastric mucosa is constantly exposed to exogenous substances, some of which, such as ethanol, nicotine and non-steroidal anti-inflammatory drugs (NSAIDs), can affect its integrity. Therefore, the mucosa possesses various protective mechanisms including the release of gaseous mediators such as NO and H_2_S, generally recognized to be implicated in the maintenance of gastrointestinal integrity and in the mechanism of gastroduodenal protection (Magierowski et al., 2015).

In this regard, GI ulceration and bleeding associated with the use of conventional NSAIDs still represent the major issues to overcome. Considerable efforts have been done in the recent past to develop innovative anti-inflammatory and analgesic drugs with minor side effects.

ATB-346 [2-(6-methoxy-napthalen-2-yl)-propionic acid 4-thiocarbamoyl-phenyl ester] (Table 1) is an H_2_S-releasing derivative of naproxen (Wallace et al., 2010). Naproxen is one of the most used NSAIDs, since some evidence suggested that its use might be associated with fewer cardiovascular side effects than selective COX-2 inhibitors and other NSAIDs. The molecule contains a 4-hydroxyphenyl-thioamide (Martelli et al., 2013), an H_2_S-releasing moiety used for the first time with this purpose. Activity of ATB-346 was evaluated in healthy animals, in several models characterized by impaired mucosal defense and in a model of gastric ulcer healing. In terms of gastric injury, ATB-346 showed to be approximately 100-fold safer than naproxen in healthy animals, exerting also comparable or superior effects respect to those of naproxen in two models of inflammation. Additionally, in opposition to selective COX-2 inhibitors, ATB-346 did not produce significant gastric damage in rats with compromised mucosal defense; it also enhances healing of pre-existing gastric ulcers. Importantly, ATB-346 exhibited a better cardiovascular profile than conventional NSAIDs.

Similarly, ACS14 [2-acetyloxybenzoic acid 4-(3-thioxo-3*H*-1, 2-dithiol-5-yl) phenyl ester] (Table 1) is a H_2_S releasing compound derived from the conjunction of acetylsalicylic acid with ADT-OH (Liu et al., 2012). ACS14 proved to be an effective and safe molecule, with significant advantages if compared to the native drug. Kinetic and metabolic experiments have shown that, after *in vivo* administration, ACS14 is transformed mostly by generating salicylic acid and ADTOH, the H_2_S-donating moiety. In addition to maintaining the thromboxane-suppressing activity, the aspirin-H2S releasing hybrid preserves the gastric mucosa integrity through the increase of H_2_S/GSH formation, thus affecting the redox imbalance processes (Sparatore et al., 2009). Additionally, a recent study revealed that ACS14 is also able to protect gastric mucosa against the aspirin induced damage through the inhibition of OS and a stimulation of local blood flow, maybe due to the involvement of K_ATP_ channels (Sun et al., 2017a).

As regards the smooth muscle physiology in GI system, H_2_S demostrated beneficial effects since H_2_S is able to inhibit muscle contraction, via the inhibition of Rho kinase and PKC activities and stimulation of MLCP activity, which lead to dephosphorylation of the 20-kDa regulatory light chain of myosin II (MLC_20_) (Nalli et al., 2015).

Different studies investigated the effect of H_2_S on nociception in GI. Even though endogenous H_2_S produced by CBS seems to contribute to visceral hypersensitivity in rats (Xu et al., 2009), exogenous H_2_S is able to decrease colorectal distension-induced nociception via activation of ATP-dependent K^+^ (K_ATP_) channels. Anti-nociceptive effects of H_2_S seem to involve also an AKT-dependent transactivation and internalization of μ-opioid receptors (Distrutti et al., 2010).

These results led to the hypothesis that the combination of an opioid receptor agonist (such as trimebutine) with an H_2_S-releasing moiety could theoretically provide an additive or even synergistic analgesic effect. Therefore GIC-1001 (Table 1), a new and improved trimebutine derivative able to release H_2_S *in vivo*, has been developed: this drug is a salt composed of trimebutine functionalized with the H_2_S-releasing counter ion 3-thiocarbamoylbenzoate (3TCB) (Cenac et al., 2015). GIC-1001 has been tested in mouse model of colorectal distension. It showed to significantly reduce, in a dose-dependent manner, nociceptive response to all injuries; this effect is considerably improved if compared to equimolar administration of its parent trimebutine maleate salt. Taking into account the better efficacy in animal model and the safety for its use in humans (phase I study completed; ClinicalTrials.gov Identifier: NCT01738425), GIC-1001 was subjected to phase II study, demonstrating a clinically significant pain reduction (ClinicalTrials.gov Identifier: NCT01926444).

Summarizing, the H_2_S release evoked by GIC-1001 potentiates the μ-opioid receptor agonistic activity of trimebutine in mouse model. Therefore, the orally administered drug salt GIC-1001 represents an alternative to i.v. sedation during full colonoscopy. Moreover, GIC-1001 could also be potentially useful as a new treatment for the irritable bowel syndrome (IBS) associated to the visceral hypersensitivity.

To date, further studies are still required to shed light on how better exploit H_2_S polyhedral activity at the GI level.

## Hydrogen sulfide and effects induced on CNS

A rapid increase in the knowledge on H_2_S biological functions suggests that defects in H_2_S metabolism may be involved in CNS diseases (Ji et al., 2017; Shefa and Yeo, 2017). These amount of data prompted many researchers to deeply investigate the pharmacological effects of this gasotransmitter as neuromodulator, neuroprotective and anti-inflammatory agent.

The primary physiological source of H_2_S in the brain is CBS, a cytoplasm Pyridoxal-5′-phosphate (PLP)-dependent enzyme (Abe and Kimura, 1996). Noteworthy, in the brain of Alzheimer's disease (AD) patients has been observed a dramatic decrease of CBS activity and a consequent severe reduction in H_2_S levels (about 55%) (Eto et al., 2002a). Likewise, endogenous H_2_S production was found to decrease during the development of Parkinson disease (PD) (Kida et al., 2011). In CNS, H_2_S, at physiological concentrations, acts mainly as neuromodulator (Kimura, 2002; Zhang and Bian, 2014) via the involvement of at least two classes of ionotropic glutamate receptors, *N*-methyl-D-aspartate (NMDA) and α-amino-3-hydroxy-5-methyl-4-isoxazolepropionic acid (AMPA) receptors, which play critical roles in synaptic plasticity (Eto et al., 2002b; Kimura, 2002). Even if the mechanism of action need to be further elucidated, H_2_S seems to acts both directly on NMDA receptor (via sulfhydrating cysteine residues) (Kimura, 2013) and indirectly, through the regulation of intracellular Ca^+2^ levels (Nagai et al., 2004).

Further studies showed that H_2_S provides protection to neurons against OS in both extracellular and intracellular microenvironments. H_2_S protects the brain through the enhancement of γ-glutamylcysteine synthetase (γ-GCS) activity and cystine transport, which lead to an increase of intracellular glutathione (GSH) levels (Kimura and Kimura, 2004). Another mechanism involved in cytoprotection is the stabilization of membrane potentials. Kimura et al. demonstrated that K_ATP_ and Cl^−^ (CFTR) channels are activated by H_2_S as protective mechanism from OS in an immortalized mouse hippocampal cell line (HT22) (Kimura et al., 2006).

H_2_S has also been found to exert both pro- and anti-inflammatory effects. It is now clear that microglia and astrocytes contribute to neuroinflammatory processes through the production of ROS and pro-inflammatory mediators. Notwithstanding, Lee et al. showed that, in presence of H_2_S, this process could be inhibited. It seems that H_2_S narrows the release of pro-inflammatory factors, including tumor necrosis factor-alpha (TNF-α), IL-1β, and nitric oxide (NO), and, at the same time, up-regulates the production of anti-inflammatory cytokines such as IL-4 and IL-10 (Lee et al., 2010; Huang et al., 2016).

Additionally, H_2_S may regulate neuroinflammation through the inhibition of LPS-stimulated inducible NO synthase (iNOS) and p38 mitogen-activated protein kinase (p38-MAPK) signaling pathways (Hu et al., 2007; Liu et al., 2015).

As a whole, these findings corroborate the functional involvement of H_2_S in neurodegenerative diseases (He et al., 2016; Yuan et al., 2017). Therefore, the restoration of correct levels of endogenous H_2_S is an appealing challenge for the development of new potential therapies for CNS disorders. Although the advantages of multi-target strategy are clear, the discovery of new multi-target drugs endowed of H_2_S-releasing properties is still in its infancy, at least in the field of neurodegenerative diseases.

A recent paper of Egea et al. (2015) described the first multitarget compound provided of neuroprotective and antioxidant properties. The molecule, named ITH12674 (Table 1), was synthesized through the combination of two pharmacophore moieties belonging to sulforaphane and melatonin. Sulforaphane is part of many members of the Brassicaceae family, such as cabbages and broccoli, and it showed a significant neuroprotective profile in OS models of neurodegenerative diseases such as AD, PD, and inflammation (Innamorato et al., 2008). The neuroprotective effect of sulforaphane, which bears an isothiocyanate function, is due to an Nrf2-mediated antioxidant response (Tebay et al., 2015). Melatonin is an endogenous whose levels decreases with aging. The neuroprotection elicited by melatonin is mainly related to its potent antioxidant and scavenger activity (Reiter et al., 2009). Thus, ITH12674 was obtained by the replacement of the amine-group of melatonin with the isothiocyanate of sulforaphane. *In vitro* assays showed that ITH12674 elicited better neuroprotective effects when compared to parent drugs (Egea et al., 2015).

The main drawback in the administration of gaseous H_2_S is the difficulty to ensure an accurate dosage thus avoiding the risk of overdose (with dramatic consequences due to H_2_S toxicity). Therefore, the development of “sulfide-precursors” or “prodrugs” able to produce H_2_S as a result of endogenous metabolism, is currently being the most investigated strategy. Actually, medicinal chemistry is exploring new natural (Citi et al., 2014) and synthetic (Martelli et al., 2013; Zheng et al., 2016; Barresi et al., 2017; Zhao et al., 2017) H_2_S-releasing agents as worthwhile tools for the development of new MTDL with H_2_S-related pharmacological properties.

As well as Brassicaceae, *Allium sativum* is another natural source of organic sulfur-containing compounds. In particular, *S*-Allylcysteine (SAC), one of the major water-soluble transformation product from garlic, turned out to be active in preventing the damage associated with OS (Rojas et al., 2011) and cancer (Nicastro et al., 2015). Moreover, it elicited cardioprotective effects in a rat model of acute myocardial infarction (Chuah et al., 2007). However, it still remains uncertain whether SAC acts as a H_2_S precursor or as a pharmacological activator of H_2_S-synthesized enzymes (Guo et al., 2013).

Recently, a structural analog of SAC, S-propargyl-cysteine (SPRC) (ZYZ-802), has been identified as a new sulfur-containing amino acid (Wang et al., 2009). SPRC represents a new H_2_S-donor agent able to reduce deleterious effects of OS since it showed to be able to prevent the decrease of H_2_S levels in rat hippocampus subjected to lipopolysaccharide (LPS) insult and inhibit TNF-α, TNF-α receptor 1 (TNFR1), and Aβ generation (Gong et al., 2011).

Santos et al. explored a set of natural-based hybrids obtained by the conjunction of SAC or SPRC moiety with tacrine, the first cholinesterase inhibitor approved for the treatment of AD. Among the new series of compounds synthesized, Tacrine-SPRC (Table 1) showed to prevent Aβ-induced toxicity and H_2_O_2_-induced OS in SH-SY5Y cells (Keri et al., 2016). Even though Tacrine-SPRC was synthesized aiming to develop a hybrid molecule between a native drug and a natural antioxidant compound, SPRC could be classified as a H_2_S-releasing agent, thus leading us to consider Tacrine-SPRC as promising scaffold for the development of new H_2_S-releasing/cholinesterase inhibitor drugs for AD therapy.

## Conclusions

Latest investigations focused on pleiotropic activity of endogenous H_2_S led to recognize it as a key mediator implicated in many physiological aspects in human body.

The multiple effects induced by H_2_S resulted from the wide variety of targets involved. Despite the efforts, the mechanisms of action that should clarify the pharmacological effects are still not completely unrevealed. Nevertheless, many attempts in exploiting the multifaceted profile of this molecule have already been accomplished by medicinal chemists and several chemical entities containing an H_2_S-releasing moiety have been synthetized.

Herein, we focused on recent investigations on multitarget molecules able to release H_2_S. A drug that potentially “hits” more that one targets offers the possibility to increase the efficacy, limiting at the same the potential drawbacks generally arising from monotherapy with a single-target drug or a combination regimen of multiple drugs. Even if a new (H_2_S-releasing) hybrid could show improved PD and PK properties than “native drug,” the main drawback for this kind of combination is to foresee a “tissue-specific” H_2_S-release and thus avoiding systemic effects. The strategy discussed here revealed that the right combination of a “native drug” with moieties structurally able to release H_2_S could improve the toxicity profile (i.e., alendronate) or ameliorate the pharmacological effects of “old” drugs (i.e., tacrine-SPRC).

Even though this mediator has been widely investigated in the cardiovascular field, recent efforts in the search of new chemical structures to control H_2_S release are paving the way to the exploitation of this pleiotropic gasotrasmitter in other therapeutic fields, such as bone diseases and neurodegenerative disorders.

## Author contributions

SS and GN contributed equally. SS, GN, and SR wrote the manuscript. RP and MM revised the content of the work. SR conceived the idea. All authors agree to be accountable for the content of the work.

### Conflict of interest statement

SR is the inventor of one patent discussed in this work. The other authors declare that the research was conducted in the absence of any commercial or financial relationships that could be construed as a potential conflict of interest.
